# Tick-Borne Co-Infections: Challenges in Molecular and Serologic Diagnoses

**DOI:** 10.3390/pathogens12111371

**Published:** 2023-11-20

**Authors:** Santiago Sanchez-Vicente, Rafal Tokarz

**Affiliations:** 1 Center for Infection and Immunity, Mailman School of Public Health, Columbia University, New York, NY 10032, USA; ss5052@cumc.columbia.edu; 2 Department of Epidemiology, Mailman School of Public Health, Columbia University, New York, NY 10032, USA

**Keywords:** tick-borne co-infections, Lyme disease, babesiosis, anaplasmosis, next-generation sequencing, capture sequencing, serology

## Abstract

Co-infections are a poorly understood aspect of tick-borne diseases. In the United States alone, nineteen different tick-borne pathogens have been identified. The majority of these agents are transmitted by only two tick species, *Ixodes scapularis* and *Amblyomma americanum*. Surveillance studies have demonstrated the presence of multiple pathogens in individual ticks suggesting a risk of polymicrobial transmission to humans. However, relatively few studies have explored this relationship and its impact on human disease. One of the key factors for this deficiency are the intrinsic limitations associated with molecular and serologic assays employed for the diagnosis of tick-borne diseases. Limitations in the sensitivity, specificity and most importantly, the capacity for inclusion of multiple agents within a single assay represent the primary challenges for the accurate detection of polymicrobial tick-borne infections. This review will focus on outlining these limitations and discuss potential solutions for the enhanced diagnosis of tick-borne co-infections.

## 1. Introduction

Over the past five decades, a wide range of ecological and human factors have resulted in a dramatic increase in the incidence of tick-borne diseases (TBDs) worldwide. A key feature in this TBD surge has been the expansion of clinically relevant tick species that has been proposed to be, at least in part, driven by climate change [1,2,3,4,5,6,7,8]. This climate phenomenon may also influence the extent and duration of outdoor activity in at-risk human populations resulting in an increased rate of tick exposure [9]. Another key aspect in the rise in TBDs has been the introduction and wider scale employment of enhanced diagnostic tools. These assays, in turn, have improved tick surveillance, leading to the discovery of new agents, and provided key insights into disease-risk assessments through the analyses of pathogen infection rates in ticks. At the beginning of the 1980s, only a handful of tick-borne pathogens were recognized in the United States (US), while currently there are 19 bacterial, viral, and protozoan microbes that have been linked to human disease, with this number likely to increase in the future [10]. These pathogens are typically found in specific geographical areas within the range of just a few tick species that serve as primary vectors of these agents to humans. A greater understanding of pathogen distribution and their prevalence in vector ticks has consequently spurred improvements in TBD diagnosis. Nonetheless, multiple aspects of the transmission dynamics of tick-borne pathogens and the corresponding illnesses are poorly understood. One such facet is the co-infections that can result from the bite of an individual tick infected with multiple pathogens. Although relatively rare in comparison to single-agent infections, co-infections can present complications in clinical and laboratory diagnosis and can potentially augment disease severity [11,12,13]. The lack of comprehensive tools targeting a wide range of agents along with a variable or transient microbial presence in the blood are among the primary limitations in the detection of co-infections. Since patients are rarely tested for the full range of agents, co-infections can result in a lack of accurate diagnosis and lead to inadequate treatment. Another challenge in co-infection diagnosis is the difficulty in determining if patients were infected simultaneously or serially with multiple pathogens [14]. Although serological studies indicate that antibodies to multiple tick-borne co-infections are not uncommon in patients with TBDs, there currently is a paucity of studies examining the incidence and the pathophysiological effects of concomitant infections in humans [15,16,17]. Here, we will review the challenges in tick-borne disease diagnosis and outline how intrinsic assay limitations can have a major role in the accuracy of the detection of polymicrobial tick-borne infections. The focus will be on agents transmitted by *Ixodes scapularis*, a species historically implicated in the majority of TBDs in the US, and *Amblyomma americanum*, a tick currently undergoing a rapid range expansion. Both tick species have been implicated as vectors of multiple pathogens and, accordingly, present a co-infection risk for humans.

## 2. *Ixodes scapularis* (the Blacklegged Tick)

Accounting for pathogen diversity and disease incidence, *Ixodes scapularis* is the key vector of zoonotic microbial pathogens in the US. Currently, this tick species is implicated in the transmission of up to seven human pathogens, although not all may circulate within the same geographical area [18]. Nonetheless, co-infections in ticks are not uncommon and surveillance studies have identified individual ticks infected with three and even up to four pathogens [19,20,21]. *Ixodes scapularis* is the primary vector of *Borrelia burgdorferi*, the agent of Lyme disease (LD), the predominant tick-borne infection in the northern hemisphere [22]. An average of 35,000 cases of LD are reported annually in the US, accounting for >80% of tick-borne infections reported by the Centers for Disease Control and Prevention (CDC) [23]. This figure may represent only a fraction of actual cases, with recent studies suggesting the actual number to be over ten-fold higher [24,25]. 

The primary factors accounting for the high incidence rates of LD in the US include the wide distribution of *I. scapularis* throughout the eastern US, frequent human exposure, as well as high tick infection rates with *B. burgdorferi*. Although tick surveillance studies have shown some geographical variation in infection rates, approximately 20% of nymphs and >50% of adult *I. scapularis* are typically infected with *B. burgdorferi* within the main geographic foci of TBDs [19,20,26,27,28] (Table 1). These infection rates are significantly higher when compared to other vector-borne pathogens found in the US. Consequently, because LD is the predominantly reported TBD, *B. burgdorferi* is typically implicated in the majority of human tick-borne co-infections. 

*Ixodes scapularis* is also the primary vector of *Anaplasma phagocytophilum,* the etiological agent of human granulocytic anaplasmosis [29] and the hemoprotozoan *Babesia microti*, the agent of human babesiosis [30]. In 2019, a total of 5655 cases of anaplasmosis and 2418 cases of babesiosis were reported to the CDC [31,32], reflecting a continual trend of increased incidence within the past two decades. The prevalence of both pathogens in ticks is lower than *B. burgdorferi*, with *A. phagocytophilum* reported in 1–9% and *B. microti* in 3–11% of *I. scapularis* nymphs [19,33,34,35,36,37,38,39] (Table 1). Dual infections of *B. burgdorferi* with *A. phagocytophilum* and *B. burgdorferi* with *B. microti* have been reported in 1–6% and 1–7% of nymphs, respectively [40,41,42]. As with all agents, both single and dual infections of *A. phagocytophilum* and *B. microti* are substantially greater in *I. scapularis* adults [19,20]. This is due to the additional blood meal acquired by this stage, resulting in a higher likelihood of infection.

**Table 1:** Pathogen prevalence in *Ixodes scapularis* and *Amblyomma americanum* ticks.

Tick Vector	Agent	Disease	Prevalence in Ticks (%)	References
*Ixodes scapularis*	*Borrelia burgdorferi*	Lyme disease	N: 10–25%, A: 40–70%	[19,20,26,27,28,43]
	*Borrelia miyamotoi*	*Borrelia miyamotoi* disease	N: 0.5–3%, A: <5%	[19,21,38,39,43]
	*Borrelia mayonii*	Lyme disease	N: 0.5–4%, A: <6%	[38,39]
	*Anaplasma phagocytophilum*	Human granulocytic anaplasmosis	N: 1–9%, A: 5–25%	[19,20,33,34,35,36,37,38,39,43]
	*Ehrlichia muris eauclairensis*	Ehrlichiosis	N: 0.5–2%, A: <3%	[38,44]
	*Babesia microti*	Babesiosis	N: 3–11%, A: 5–25%	[19,20,33,35,36,37,38,39,43]
	Powassan virus	Powassan encephalitis	N: <2%, A: <2%	[19,21,38,43]
*Amblyomma americanum*	*Ehrlichia chaffeensis*	Human monocytic ehrlichiosis	N: 1–3%, A: 5–12%	[19,45,46,47,48]
	*Ehrlichia ewingii*	Human ewingii ehrlichiosis	N: 1–3%, A: 3–8%	[19,45,46,47,48]
	Panola Mountain *Ehrlichia*	Not confirmed	N: <1%, A: <2%	[19,45]
	*Rickettsia amblyommatis*	Not confirmed	N: 15–55%, A: 40–85%	[19,47,48,49,50,51,52,53]
	*Borrelia lonestari*	Not confirmed	N: <1%, A: 1–5%	[19,45,46,47,48,54]
	*Francisella tularensis*	Tularemia	N: <0.05%, A: <0.05%	[55]
	Heartland virus	Heartland virus disease	N: <2%, A: <2%	[56,57]
	Bourbon virus	Bourbon virus disease	N: <1%, A: <1%	[56,58]

N: nymphs; A: adults.

Additional human pathogens transmitted by *I. scapularis* include a relapsing fever-like *Borrelia* species, *B. miyamotoi* [59], and Powassan virus, a rare but potentially life-threatening tick-borne flavivirus [60]. Recently, *Ehrlichia muris eauclairensis*, a human pathogen whose distribution was thought to be limited to the upper Midwest has been found in host-seeking *I. scapularis* nymphs collected in the Northeast [38,44,61]. *Borrelia mayonii*, a rare causative agent of LD has thus far only been reported within the upper Midwest [38,62,63]. These four agents are infrequently detected in tick surveillance studies with typically only a 1–3% individual pathogen prevalence in nymphs [19,21,38,39] (Table 1). This is in line with rare reports of annual human infections with these agents [64,65,66]. Nevertheless, all four pathogens have been detected as co-infecting agents alongside *B. burgdorferi*, *B. microti* and *A. phagocytophilum* in individual ticks [19,21,38].

The feeding habits of *I. scapularis* are a major factor in the large diversity of pathogens that can be acquired by this tick. The white-footed mouse, *Peromyscus leucopus,* is the predominant host for the larval and nymphal stages of *I. scapularis*, and it is also the primary reservoir of *B. burgdorferi* [67], *A. phagocytophilum* [29], *B. microti* [68], Powassan virus lineage II [69], and *B. miyamotoi* [70]. *Peromyscus leucopus* can be asymptomatically co-infected with >1 of these pathogens and, subsequently, *I. scapularis* can acquire multiple pathogens during a blood meal. For some agents, pathogen acquisition by *I. scapularis* may also be facilitated by the presence of other pathogens in the vertebrate host. Specifically, the acquisition of *B. microti* from mice may be enhanced when ticks feed on animals concurrently infected with *B. burgdorferi* [71]. Following pathogen acquisition, co-infected ticks can simultaneously transmit these agents to other animals as well as humans during subsequent bloodmeals.

The range of *Ixodes scapularis* has increased substantially in recent decades and it is now distributed throughout Eastern and Central US [72]. Although *I. scapularis* is not found on the West Coast of the US, this region is endemic to a closely related species, *Ixodes pacificus*, that can transmit some of the same pathogens as *I. scapularis* [73,74]. However, because of ecological reasons such as feeding behavior and/or host preference, *I. pacificus* is implicated in only a fraction of TBDs compared to *I. scapularis*.

## 3. *Amblyomma americanum* (the Lone Star Tick)

In recent decades, the geographic range of *A. americanum* has expanded northwards from its historical southern endemic regions [75]. This rapid expansion, coupled with this tick’s highly aggressive nature and capacity for harboring a large diversity of pathogens has positioned *A. americanum* as arguably the second most relevant tick species for human health in the Eastern US, aside from *I. scapularis*. *Amblyomma americanum* is the vector of *Ehrlichia chaffeensis* and *E. ewingii* and the *A. americanum* range expansion has concurrently been associated with an increase in the incidence and geographical expansion of human ehrlichiosis [76,77]. Another uncharacterized *Ehrlichia* species, occasionally referred to as Panola Mountain *Ehrlichia* has also been reported in *A. americanum* [78], although the pathogenicity of this agent is currently unknown (Table 1).

*Amblyomma americanum* is the only known vector of the Heartland virus and Bourbon virus. Infection with either virus can cause a severe life-threatening illness [58,79,80,81] (Table 1). Although originally identified in the Midwest and Southern US, recent detection of these viruses in nymphs collected in the Northeast suggests a much wider geographical distribution [82,83,84].

Southern tick-associated rash illness (STARI), an inflammatory skin condition, has also been linked with *A. americanum* [85]. At present the etiology of STARI remains uncertain and there is currently no evidence linking STARI to any microbial agent [86]. One proposed, but ultimately disproved agent of STARI was a spirochete *Borrelia lonestari* which can be present in 1 to 5% of *A. americanum* [19,45,47,54,87,88,89] (Table 1). Although not directly implicated in human disease, a recent report suggests *Borrelia lonestari* may on occasion be an opportunistic pathogen [90].

*Amblyomma americanum* has also been implicated in the increase in the incidence of mild spotted fever group rickettsiosis (SFGR) [91]. The majority of *A. americanum* are infected with *R. amblyommatis* and several serosurveys and animal studies have implicated this agent as a potential cause of mild rickettsiosis [19,48,50,53,92,93,94,95,96,97,98,99] (Table 1). At present, however, its role in human disease remains controversial.

*Amblyomma americanum* has been reported as a competent vector of *Francisella tularensis* in the Southern states [55,100,101,102]. However, early studies reported that the rate of infection of *F. tularensis* in *A. americanum* was very low (<0.05%) and contemporary surveillance studies focused on *A. americanum*-associated pathogens have not targeted *F. tularensis* [19,48,53,55,103] (Table 1).

Polymicrobial infections have been reported in *A. americanum* ticks but no human co-infections with *A. americanum*-borne agents have been recorded thus far [47,104,105]. This is likely due, at least in part, to the low infection rates of these agents in *A. americanum,* coupled with lower rates of laboratory testing compared to *I. scapularis*-borne pathogens. Nonetheless, the recent population explosion of *A. americanum* throughout the Eastern US requires increased public awareness and appropriate laboratory testing to identify all potential *A. americanum* infections, especially in areas where it has become the dominant human-biting tick.

## 4. Other Clinically Relevant Tick Species

There are several other tick species endemic to North America that are implicated in pathogen transmission. Prior to the initial characterization of Lyme disease, *Dermacentor variabilis* (the American dog tick) was the major tick species associated with human disease. *Dermacentor variabilis* is the primary vector of *Rickettsia rickettsii*, the causative agent of Rocky Mountain spotted fever (RMSF) in the eastern US [106]. Over the last two decades, the incidence of SFGR has steadily increased and it is currently the most frequently diagnosed TBD, second only to LD [23,107,108]. Paradoxically, *R. rickettsii* is rarely detected in surveillance studies of *D. variabilis,* suggesting that other *D. variabilis*-borne *Rickettsia* spp. or species transmitted by other ticks might be the primary cause of the rise in SFGR [51,91,109,110]. *Dermacentor variabilis* is also the primary vector of *F. tularensis* [111]. Although *D. variabilis* has been attributed with being responsible for at least two outbreaks of tularemia in the US [112,113], the infection rate of *F. tularensis* in field-collected *D. variabilis* has been estimated to be <1% [114]. *Rhipicephalus sanguineus* (the brown dog tick), has also been implicated in the transmission of *R. rickettisi* in the southwestern US and along the US–Mexico border [115]. Another *Amblyomma* species, *A. maculatum* (the Gulf Coast tick) is widely distributed in the southeastern and south-central United States and has recently been found in the Northeast [116,117,118,119]. This species is the main vector of *R. parkeri,* another agent implicated in SFGR, with infection rates of up to 56% in questing adult ticks. [120]. These tick species, along with *Dermacentor andersoni*, *D. occidentalis*, *Ixodes cookei*, and *Ornithodoros* spp., all contribute to the spectrum of TBDs in the US. However, these ticks are typically implicated in the transmission of only a single human pathogen, making them less relevant for discussions of co-infections.

## 5. Co-Infections in Patients with TBDs

Although tick infections with multiple agents are not uncommon, reports describing human tick-borne co-infections are rare. This is likely influenced by a combination of several key factors, such as the variable transmission dynamics of tick-borne pathogens, diagnostic assay limitations, lack of testing and subclinical infections. Co-infections, when recorded, are typically identified in LD patients, and include either *B. microti* or *A. phagocytophilum,* undoubtedly because of the high infection rates of these pathogens in *I. scapularis* [121]. However, there is an overall lack of data correlating tick co-infection rates and co-infections in patients with TBDs. Part of the difficulty is that prevalence in ticks may often not be a clear indicator of the rate of transmission to humans. For example, the prevalence of infectious *A. phagocytophilum* in ticks tends to be misrepresented, as surveillance studies typically do not differentiate between strains that are infectious or non-infectious to humans. In addition, certain rare pathogens such as Powassan virus can be transmitted in only 15 min from the tick to a vertebrate host [122]. Conversely, other, more prevalent agents, may only be transmitted after >24 h of tick attachment [123,124].

The limited number of studies that focused on co-infections in TBD patients show little congruence and were based on serologic data. Putative co-infections with *B. burgdorferi* and *B. microti* were reported in 2–27% of patients [13,16,125,126], whereas potential co-infections with *B. burgdorferi* and *A. phagocytophilum* ranged from 2 to 15% [126,127]. In addition, few studies investigated the disease severity that can result from a co-infection, with discrepant outcomes [128]. In one study, patients co-infected with *B. burgdorferi* and *B. microti* developed a more severe illness during the early stages of disease and required longer recovery time than single-infected patients [13]. Another study, however, concluded that co-infection with both agents did not have an impact on disease severity [129]. The latter study examined the presence of antibodies to either *B. burgdorferi* or *B. microti* along with clinical diagnoses and could not clearly delineate if the infections were concurrent or occurred separately, which is a major intrinsic limitation of antibody-based analyses. The few studies that investigated the severity of *B. burgdorferi* and *A. phagocytophilum* co-infections in humans were also conflicting. One study showed that co-infected patients were more likely to be symptomatic [128], whereas in another study the array of symptoms in co-infected patients was not significantly different from patients with only LD [130]. This discrepancy was addressed by Horowitz and colleagues who reported that the criteria used to define *A. phagocytophilum* co-infection in LD patients were a factor in disease severity, and that patients presenting with a genuine co-infection recorded significantly more symptoms than patients with only LD [17].

There have been relatively few animal model studies examining tick-borne co-infections, also with contradictory outcomes. In a study addressing the co-infection of *B. burgdorferi* with *B. microti*, the authors concluded that both agents followed an independent course of infection after observing no statistical significance in arthritis and carditis pathology between single and co-infected mice [131]. Conversely, other studies determined that arthritis severity was significantly higher in co-infected mice than in mice infected solely with *B. burgdorferi* [132,133]. Simultaneous co-infection with *B. burgdorferi* and *A. phagocytophilum* appears to enhance the pathogenesis of LD in laboratory mice due to the ability of *A. phagocytophilum* to functionally damage neutrophils which play a key role in the early defense against infection of *B. burgdorferi* [11,12].

## 6. Laboratory Diagnosis

### 6.1. Culture and Microscopy

Isolation of the pathogen in culture is still considered the gold standard in laboratory diagnosis [134,135,136]. Because of its clear lack of utility for rapid diagnosis coupled with the large resource requirements, the culture diagnosis of tick-borne diseases is impractical, and this method is not widely used in clinical laboratories. In addition, culture isolation can often be unsuccessful, even from positive specimens [137,138], while some agents such as *E. ewingii* are not cultivable [139,140]. Even when culture is successful, co-infections cannot be identified by a single method, as each agent typically requires its own culture media and/or cell lines [139,141,142]. For example, Barbour–Stoenner–Kelly (BSK-H) medium used for *B. burgdorferi* isolation is unsuitable for the isolation of obligate intracellular microbes such as *Babesia* or *Anaplasma*. Thus, culture is generally only useful as a confirmatory diagnostic tool or in the event that other more rapid diagnostic assays fail.

Visual analysis of Wright- or Giemsa-stained peripheral blood smears by microscopy is typically the quickest diagnostic method for the identification of intracellular tick-borne infections with *B. microti*, *A. phagocytophilum*, and *Ehrlichia* spp. [135,143]. However, this method is unsuitable for the detection of Powassan virus as well as *B. burgdorferi*, where the low numbers of spirochetemia are highly unlikely to be visualized. In addition, examination of blood smears is labor intensive and requires specially trained personnel as the identification of the intraerythrocytic pleomorphic ring-shaped *Babesia* trophozoites and the *Anaplasma*/*Ehrlichia* morulae within monocytes can be challenging. This method also cannot differentiate between *A. phagocytophilum* and *E. ewingii* as both agents infect neutrophils [144]. Typical of all diagnostic tests, the sensitivity is highly dependent on the level of bacteremia/parasitemia and the time after the onset of clinical illness. Blood smear evaluation is relatively sensitive for anaplasmosis because 25 to 75% of reported cases have observable morulae in peripheral blood, and sensitivity is the highest during the first week of infection. Conversely, microscopy is not useful for detection of *E. chaffeensis* since morulae are present in only <10% of infected cases during the acute stages of disease [139]. For the diagnosis of babesiosis, microscopical evaluation has poor sensitivity in patients with low levels of parasitemia in the early or chronic stages of disease and requires multiple smears over a period of days to confirm the diagnosis [145]. Moreover, microscopy enables only genus-level identification and cannot discriminate between *Babesia* from *Plasmodium* parasites [146], although this problem is rarely encountered in the US.

Microscopy is not very useful for the detection of co-infections. Aside from the aforementioned lack of utility in detecting *B. burgdorferi* and Powassan virus, visualizing other co-infections using this approach is difficult due to variable microbial growth rates. For example, both *A. phagocytophilum* and *B. microti* have different incubation periods of up to 2 and 4 weeks, respectively [147,148]; therefore, both agents may not be simultaneously present at a visible, detectable rate within a single blood smear.

### 6.2. Molecular Methods

Polymerase chain reaction (PCR) is the primary molecular assay used for the clinical diagnosis of TBDs [143,148,149,150,151]. The main advantages of PCR over culture and microscopy are its low cost, speed, and labor. The primary limitations are the requirement for matching primer sequences and template material, which can inhibit the detection of new strains and prevent detection of novel agents.

The sensitivity of PCR varies depending on the agent targeted, the sample type, and the timing of specimen collection (acute vs. convalescent infection) [152,153]. Whole blood is the most frequent specimen used in molecular testing and a PCR of whole blood is useful for the diagnosis of acute anaplasmosis and babesiosis [143]. The PCR sensitivity for patients who are culture positive for *A. phagocytophilum* is approximately 80%, whereas in patients who have a positive blood smear for *B. microti,* the sensitivity level increases to up to 100% [145,154]. The sensitivity of PCR in patients with human monocytic ehrlichiosis has been reported to range between 56 and 100%, depending greatly on the immune response of the patient. Negative or low antibody titers in the acute-phase serum samples correlated with high PCR sensitivities of 87–100%, whereas the sensitivity of PCR decreased to 56% in patients with high antibody titers, suggesting that a low bacteremia might be influenced by a well-established immune response [155,156,157].

PCR assays are also used for *Borrelia miyamotoi* disease (BMD) and rickettsial diseases [59,151]. For *Rickettsia*, PCR analyses of whole blood can be useful for detection during the acute stage. However, assay sensitivity may be affected by the low number of rickettsiae present in the blood, as the majority of *Rickettsia* are typically present in the vascular endothelium [158]. Additionally, current molecular assays may not identify rickettsiae up to species level which limits epidemiological insights [159,160].

A major limitation of PCR in TBD diagnosis is its lack of utility for the detection of *B. burgdorferi* in the blood. Low and transient spirochetemia in the early acute stage of disease results in very poor sensitivity [161]. A notable exception is *B. mayonii* where a higher level of spirochetemia is typically seen in the early stages of the disease [63]. Sensitivity improves drastically when testing other sample types, including EM lesions [161] and synovial fluid [162,163], although it is not useful for testing the cerebrospinal fluid of patients with neuroborreliosis [134]. Similarly, PCR assays are used to detect viral RNA on serum or cerebrospinal fluid specimens collected during acute tick-borne viral infections, but because of transient viremia, the diagnostic performance of these assays is highly constrained by the timing of the sample collection [164,165].

Another limitation of PCR is that most assays test for only a single agent, thus preventing the detection of co-infections. To overcome this shortcoming, multiplex PCR assays have been developed that can test for several tick-borne pathogens in a single assay [166,167,168,169]. This approach simplifies TBD testing and has even led to the discovery of novel tick-borne pathogens [38,62,170]. Despite their utility, multiplex PCR assays also have some key limitations. Most assays target only three to five agents, representing a considerable shortcoming in diagnostic capacity when employed within a TBD endemic area with a wide spectrum of co-circulating agents [21,43,47,48,62,168,171]. In addition, as with standard PCR, multiplex assays require matching primer sequences and template material and thus are unlikely to detect new emergent agents.

### 6.3. Next-Generation Sequencing (NGS)

Within the past decade, NGS has moved from a fringe and complex niche technique to one that has gained acceptance in laboratories worldwide and revolutionized biomedical research. NGS is now used for a wide range of applications, including genomics, epigenetics, transcriptomics, oncology, personalized genome medicine, and pathogen detection and discovery. During this time, technological advancements have led to a decrease in NGS costs, labor, and the length of time required for data generation and analyses. NGS has also begun to gain prominence in clinical microbiology and public health.

Unbiased next-generation sequencing (UNGS) methods are better suited than PCR for the molecular detection of diverse microbial pathogens and are unapparelled for the detection of polymicrobial infections. Whereas PCR requires precision in matching the sequences of primers and reporter oligonucleotides with those of templates, there is no such requirement with UNGS, where random oligos facilitate the amplification of all nucleic acids in the sample. NGS data is analyzed through BLAST homology searches of every amplified product, irrespective of species origin. As a result, UNGS can simultaneously detect the whole spectrum of agents within a single sample and identify novel species or strains [172,173,174,175,176]. PCR still retains advantages in sensitivity, cost, and simplicity due to the substantial level of infrastructure needed for NGS employment. Nonetheless, several improvements in NGS have substantially reduced operating costs. These include the introduction of streamlined bioinformatic analysis pipelines, and the employment of dual index barcoding that facilitates the examination of >50 samples within a single assay [177,178].

NGS techniques have been indispensable for the characterization of the tick microbiome studies. Prior to NGS studies, the understanding of the tick bacteriome was limited, while the awareness of the tick virome diversity was nearly non-existent. Over the past decade, NGS studies provided key insights into the diversity of tick-associated viruses and other microbes [28,179,180,181,182]. In *I scapularis* alone, the unbiased nature of NGS facilitated the identification of over 15 novel viral sequences, whereas before, POWV was the lone virus known to be associated with this tick species [183,184].

Ideally, TBD diagnosis using NGS-based platforms would provide much-needed broad range analyses of clinical specimens. Thus, NGS-based diagnostics would present an advance beyond the confines of targeted assays like PCR and would theoretically provide a single test for all microbes. Genetic polymorphisms associated with new strains or species that may be missed by PCR assays would be rendered irrelevant and readily detected in NGS due to the unbiased nature of random amplification. Nonetheless, despite its vast potential, there are several limitations of NGS that need to be addressed before wide-scale employment of NGS for diagnostics. Sensitivity is a major shortcoming of UNGS, especially when compared to PCR, with specific amplification associated with PCR resulting in considerably greater sensitivity over random amplification of UNGS. In addition, the establishment of clear and reproducible positivity thresholds can be challenging and remains one of the primary obstacles for the deployment of this technique for diagnostics.

**Figure 1:**
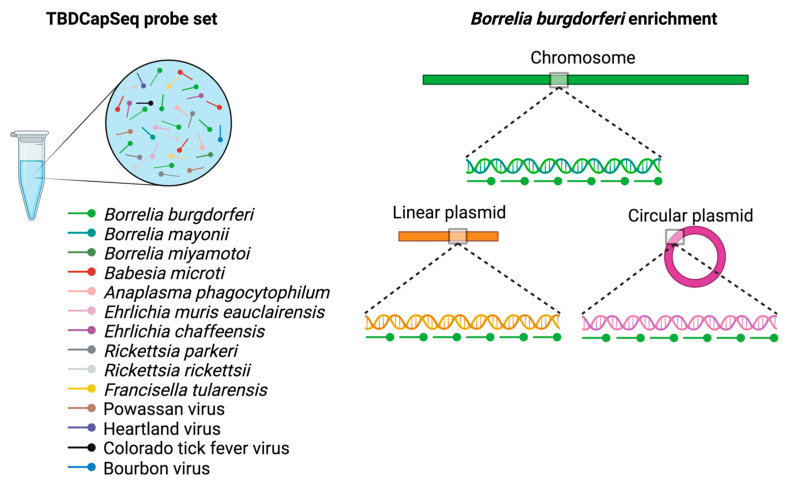
TBDCapSeq probe design. The probe mix targets 14 tick-borne pathogens found in the US. For each pathogen, the capture probes are designed along the entire length of each genomic segment. The probes, shown in red, are approximately 100 nucleotides (nt) in length and bind to a region within 50 to 100 nt from its next nearest genomic neighbor, resulting in thousands of probes per agent.

One approach for addressing the sensitivity limitations of UNGS is through the employment of capture sequencing. This NGS-based approach results in a highly sensitive and multi-agent inclusive assay that provides advantages over both PCR and UNGS for pathogen detection. Capture sequencing uses agent-specific probes to selectively capture the template of interest prior to sequencing, resulting in a substantial increase in sensitivity that surpasses PCR [185,186]. Although it is a targeted approach, it does not have the stringency of PCR and can include probes for a wide range of agents (Figure 1).

**Figure 2:**
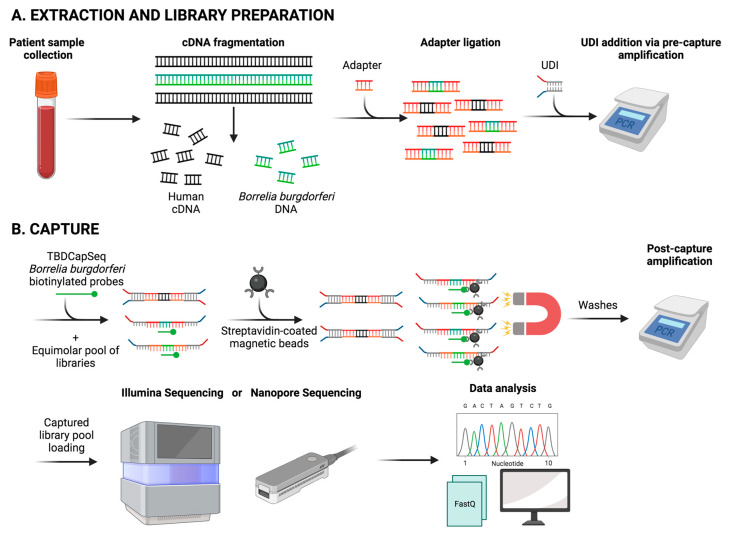
Schematic of TBDCapSeq. The workflow consists of DNA library preparation, capture of target DNA libraries and NGS sequencing. (**A**) For the library preparation, nucleic acid is extracted from a clinical sample and enzymatically sheared into short fragments (<200 bp). Fragmented DNA is ligated at both ends with universal adaptors to generate dual-indexed libraries. (**B**) Amplified libraries are pooled for hybridization with custom biotinylated TBDCapSeq enrichment probes. Subsequent probe-bound fragments are then captured with streptavidin beads. The probe-bound DNA is eluted and amplified using universal primers, followed by quantification and NGS.

The utility of this approach has been documented for viral and bacterial agents, and the development of the TBD capture sequencing assay (TBDCapSeq) demonstrated its superior capacity for the detection of tick-borne pathogens in ticks, rodents and clinical specimens with a sensitivity equal to, or occasionally, superior to PCR [187,188]. Capture sequencing can provide a substantial improvement over PCR for the detection of *B. burgdorferi* in blood, and it can simultaneously detect any tick-borne infection including concurrent infections [188] (Figure 2).

The high costs and size of NGS sequencers, coupled with the current assay time of >12 h present another prohibitive roadblock to NGS-based diagnostics. However, the advent of portable sequencers has created a potential way to overcome these limitations and establish NGS as a deployable frontline diagnostic platform [189]. With minimal upfront processing and the requirement of only a laptop, handheld sequencers are significantly faster and less expensive than conventional benchtop instruments. The addition of capture sequencing techniques to these versatile sequencers can enable pathogen detection and discovery at speeds unprecedented for other NGS platforms.

### 6.4. Serology

Serology remains an important tool for TBD diagnosis, but its application can vary based on the targeted pathogen and the disease progression. The most frequently used antibody-based assays for TBD diagnosis include the enzyme-linked immunosorbent assay (ELISA), indirect immunofluorescent assay (IFA) and Western blotting [143,149,190,191]. For agents with high blood bacteremia/parasitemia that are typically diagnosed by PCR or microscopy, such as *Babesia*, *Anaplasma*, *Ehrlichia,* and *Rickettsia*, serology is not the primary diagnostic approach. When serology is used, IFA on paired acute and convalescent serum samples is the standard test [135,148,191]. However, the accuracy of IFAs can vary among laboratories, primarily due to the lack of standardized antigenic targets, cross-reactivity, or the subjective establishment of positivity thresholds [151]. Serologic tests for viral agents such as Powassan virus, Heartland virus and Bourbon virus typically utilize neutralization assays [165,192]. Because of the biosafety considerations, these tests are typically only performed in Biosafety Level-3 facilities, which limits the number of laboratories that can conduct these assays [192,193].

Because molecular assays are typically not useful for the detection of *B. burgdorferi*, serology is the gold standard for the laboratory diagnosis of LD [143]. The two-tiered test algorithm (TTT) consists of an ELISA followed by either a supplemental IgG and IgM Western blot [134] or an additional ELISA [194]. Although this method is very sensitive in disseminated disease (70–100%), it is less useful during early infection (<40%) [195]. The subjective nature of the TTT may also impact reproducibility [134].

A major limitation of serologic assays is that current assays only detect antibodies against a single agent. Thus, co-infections can only be identified by the employment of multiple different agent-specific tests, increasing cost and time for accurate diagnosis. These factors, along with a lack of awareness of co-infections may result in physicians only ordering a single diagnostic test, which can have vital implications for treatment. For some co-infections, like LD and anaplasmosis, the antibiotic regimen is similar. For others, the accurate detection of co-infections is crucial for the initiation of proper disease-specific treatment regiments. For LD, doxycycline, amoxicillin, or cefuroxime are the first-choice antibiotics [196]. However, a different treatment regimen is used for babesiosis that includes a combination of atovaquone, an antiparasitic, plus azithromycin, and, for severe disease, quinine with clindamycin [143]. Thus, therapy for LD will not resolve babesiosis.

Assay specificity is another major intrinsic challenge in serology. The identification and subsequent employment of short and agent-specific immunodominant peptides represents a potential solution to problems relating to cross-reactivity and also provides an opportunity for multi-agent serologic tests. Employing these agent-specific peptides within a single assay using existing technology such as microarrays or Luminex assays would simplify serologic testing and lower costs [197,198,199,200]. The potential utility of this approach to successfully discriminate antibody responses to different tick-borne agents using microarrays has already been shown [201,202,203]. This approach can also differentiate between the IgM and IgG responses that are critical for the delineation between historical and current infections. Nonetheless, the requirement for the presence of detectable antibodies can be a prohibitive factor for the use of serology for the early detection of some TBDs. Ultimately, a coordinated effort, using a multi-agent serologic platform alongside an NGS-based molecular assay for direct agent detection may be required to provide the most accurate and broad TBD diagnosis. 

## Data Availability

No new data were created or analyzed in this study. Data sharing is not applicable to this article.
